# Clinical Performance of the Gore Septal Occluder in Patent Foramen Ovale Closure in Different Septal Anatomies: 1-Year Results from a Single-Center Experience

**DOI:** 10.3390/jcm12185936

**Published:** 2023-09-13

**Authors:** Giuseppe Verolino, Dario Calderone, Mara Gavazzoni, Davide Sala, Paolo Sganzerla

**Affiliations:** 1 Invasive Cardiology, Department of Cardiology, San Luca Hospital, IRCCS Istituto Auxologico Italiano, 20149 Milan, Italy; g.verolino@auxologico.it (G.V.); p.sganzerla@auxologico.it (P.S.); 2 Cardiology Unit, San Luca Hospital, IRCCS Istituto Auxologico Italiano, 20149 Milan, Italy

**Keywords:** patent foramen ovale, complex PFO anatomy, GSO, long-term residual shunt

## Abstract

Background: PFO (Patent foramen ovale) is a common defect that affects about 25% of the population. Although its presence is asymptomatic in the majority of the cases, the remaining part becomes overt with different symptoms, including cryptogenic stroke. PFO closure is currently a widely available procedure in complex anatomy, with Amplatzer PFO Occluder (APO) being the most commonly used tool. However, the performance of another device, the GORE Septal Occluder (GSO), has not been completely explored with regard to different septal anatomies. Methods: From March 2012 to June 2020, 118 consecutive patients with an indication of PFO closure were treated using the GSO system, included in a prospective analysis, and followed. After 12 months, every patient underwent transcranial Doppler ultrasound to evaluate the effectiveness of treatment. Results: Of 111 patients evaluated, 107 showed effective PFO closure (96.4%), and 4 showed a residual shunt (3.6%). To better evaluate the device performance, the overall population was sorted into two clusters based on the echocardiographic characteristics. The main difference between groups was for PFO width (4.85 ± 1.8 vs. 2.9 ± 1 mm, *p* < 0.001) and PFO tunnel length (12.6 ± 3.8 vs. 7.2 ± 2, *p* < 0.001), allowing complex and simple anatomies to be identified, respectively. Regardless of the aforementioned cluster, the GSO performance required to reach an effective closure was independent of anatomy type and the chosen device size. Conclusion: The GSO device showed a high closure rate at 1-year follow-up in patients, with at least one anatomical factor of complexity of PFO irrespective of the level of complexity itself.

## 1. Introduction

Patent foramen ovale (PFO) is involved in several types of disease and pathological situations, and in recent years, the popularity of closure procedures and devices has been challenged by concerns due to the increasing evidence of long-term complications [1,2,3].

Currently, the procedure of PFO closure should be performed after multidisciplinary evaluation, carefully considering the balance between risk and benefit. Furthermore, an accurate imaging evaluation of septal anatomy is pivotal because several factors are demonstrated to be associated with a higher risk of relapsing ischemic events and a higher risk of complications [4].

Several devices for percutaneous PFO closure have been developed, and the optimal prosthesis should be that one provides the following: (i) effective PFO closure, (ii) simplicity in the procedure for the implant, (iii) suitability for every anatomical situation, and complexity of septum, (iv) low interference with other intracardiac structures, (v) possibility to be removed if required, and (vi) a low incidence of complications. Each of the available devices fits only one or more of these characteristics; therefore, the choice of the device should depend on the experience of the center and on the basic characteristics of the manufacturer. Currently, the most broadly used prosthesis is the Amplatzer Occluder (Abbott Vascular), which is beneficial for clinical randomized studies and easy to implant; however, its almost rigid structure raises some concerns about device erosion and interference with venous surrounding structures and valvular function [1,2,3].

To overcome this issue, the Gore Helex occluder (Gore Medical, Flagstaff, Arizona) was developed as a single-wire nitinol structure covered with polytetrafluoroethylene (ePTFE) that provides greater conformability and a good apposition to septal structures [3]. After initial complications [5,6], consistent modifications of the delivery system and technical characteristics were carried out, and the second-generation Gore Septal Occluder (GSO; Gore Medical) replaced the first-generation model. Full details of the device are described elsewhere [7]. With its low device profile and relatively simple procedure, GSO showed its efficacy and safety in the first clinical series [7,8] and confirmed these factors in a larger randomized trial [9].

Despite this huge experience, thus far, no precise demonstration of its performance in different real-life anatomical settings of the interatrial septum has been made in order to assess the advantages of Gore better. This study aims to present a prospective single-center experience with GSO for PFO closure and to define which anatomical characteristics of the included population are related to anatomical and clinical results at mid-term follow-up.

## 2. Materials and Methods

### 2.1. Patient Population and Imaging

Between March 2012 and June 2020, all consecutive patients with an indication of PFO closure treated using the GSO system in a single Italian center were included in a prospective analysis and follow-up. The device choice was made based on the operator’s preference. The patients were treated at the department of cardiology, whereas the diagnosis of cryptogenic stroke or transient ischemic attack (TIA) was performed either by the department of neurology or the referring cardiologist or neurologist. Neuroradiologic imaging (computer tomography, CT, or magnetic resonance imaging CMR) was performed according to specific indications (suspected cerebrovascular events).

An indication of PFO closure was posed after collegial discussion and according to the most recent guidelines [4] and, thus, mainly accounted for the occurrence of one or more documented cerebral thromboembolic events related to paradoxical embolism on top of antithrombotic regimens. Before the procedure, all other embolism sources or prothrombotic factors were excluded (atrial fibrillation, pulmonary shunts, coagulation defects, active cancer, or orthopedic surgery history <3 months).

Preoperative transesophageal echocardiography (TEE) was performed by an expert operator with a high-resolution probe (TEE Multiplanar 5 Mhz probe and GE Vivid E9, GE Healthcare, Chicago, IL, USA) to establish a full morphological definition of PFO, an adequate grading of the right-to-left shunt (RLS), defined as small if there were <5 microbubbles passage, moderate if there were 5–25 microbubbles, and severe if >25 microbubbles were observed within the first 3 cardiac cycles, and plan the closure procedure [10,11,12]. The study was conducted according to the guidelines of the Declaration of Helsinki and approved by the Ethics Committee of Istituto Auxologico Italiano (protocol 01–23/10/2014). A written consensus form was administered to and signed by every patient.

### 2.2. Procedural Details

The PFO closure procedure was performed with fluoroscopic guidance, under local anesthesia and slight sedation, through a femoral vein access with a Multipurpose 5F catheter (Cordis, Hialeah, FL, USA) and 0.035 J-tip wire to cross the defect. The imaging was done with intracardiac echocardiography (ICE) through contralateral femoral vein access with 8F sheath (8 MHz ACUNAV ultrasound catheter—Biosense Webster, Johnson & Johnson, Irvine, CA, USA). After crossing the defect, a stiff wire was positioned in the left superior pulmonary vein to advance a 14F sheath and the delivery system. The Occluder device size was chosen based on the echocardiographic findings. As fully described elsewhere [7], the GSO is available in disc diameters from 20–30 mm (in 5 mm increments); it is preassembled and loaded into a delivery catheter, which is then inserted and advanced into the left atrium along the stiff guidewire by means of the preformed monorail port. Expansion of the left and then right atrial discs of the device was performed under fluoroscopy and echocardiography; the device subsequently detaches from the delivery mandril, removing tension from the system, thus allowing it to reach its final positioning with the correct orientation on the atrial septum. The device is retrievable at any stage during the delivery until it is locked to the mandril. After septal crossing, a single unfractionated heparin (UFH) bolus ranging from 5000 to 7500 UI is routinely administered. In the perioperative period, all patients received antibiotic prophylaxis (cefazolin 2 g); moreover, a double antiplatelet therapy with Acetylsalicylic Acid 100 mg/daily and Clopidogrel 75 mg/daily was started on the day of the procedure. A loading dose was administered to the patient in order to avoid any possible noncompliance with the prescription. The double antiplatelet therapy (DAPT) regimen was maintained until 6 months after the procedure; then, one antiplatelet drug was stopped, and the other was assumed lifelong. After discharge, the patients were followed with a 1-month clinical evaluation for events monitoring and a 12-month clinical and instrumental with transcranial Doppler (TCD) evaluation to identify residual relevant shunt.

### 2.3. Endpoint and Follow-Up

The main outcome measure was the presence of a residual interatrial shunt at the 12-month follow-up, detected by TCD after intravenous injection of agitated saline contrast and device size impact on the residual long-term shunt. The residual right-to-left shunt degree (rRLS) was categorized as follows: no microbubble evidence as anatomical closure, 5–10 microbubbles as functional closure, and >10 microbubbles as effective residual shunt [13].

Differences in clinical, procedural, and echocardiographic features between patients in the overall population were tested in a cluster analysis relating the echocardiographic characteristics to the main outcome.

### 2.4. Statistical Analysis

All continuous variables were presented as mean (or median) ± standard deviation (or interquartile range), and categorical variables as numbers and percentages. The normal distribution of continuous variables was evaluated with the Kolmogorov–Smirnoff test. A one-way ANOVA (with Bonferroni post-hoc correction) was elaborated to explore the difference in means; as for the association of categorical variables, the Chi-square test was used. A univariable logistic regression was made to identify the potential impact of the cluster on the residual shunt after 12 months. The two-sided statistical significance was established for *p*-value < 0.05. All statistical analyses were performed using R (R Foundation for Statistical Computing, vers 4.3.0) with the Jamovi interface and SPSS v.25 (SPSS Inc., Chicago, IL, USA) for Windows.

## 3. Results

### 3.1. Population Characteristics: Clinical and Echocardiography

Between March 2012 and June 2020, 118 patients underwent PFO closure with the Gore Septal Occluder device. All demographic characteristics are reported in Table 1.

The PFO closure indications were a TIA in 50 (42%) patients and stroke in 68 (58%). Within the overall population, 101 patients (86.1%) have a CT or CMR positive for de-novo cerebral lesion.

Before the procedure, the antiplatelet therapy consisted of ASA or Clopidogrel (SAPT) for 97 patients (82%). All the procedures were successful, and there were no relevant (defined with a right atrium contrast injection) or detectable rRLS at the end of the procedure. Table 1 summarizes all the morphological characteristics and the procedural features. To choose the most appropriate device size, TEE was carefully reviewed before each procedure in 100% of the included population. The decision whether to use a 20 mm, 25 mm, or 30 mm device was based on a qualitative assessment of the size of the ASA and the size of the PFO on preprocedural TEE. The device size ranged between 20 to 30 mm within the study population. A 20 mm was used in 5 patients (5% of the whole population), while the 25 mm was the most used in 92 patients (78%). Only in 19 patients (16%) atrial septum aneurysm (ASA) was found.

**Figure 1:**
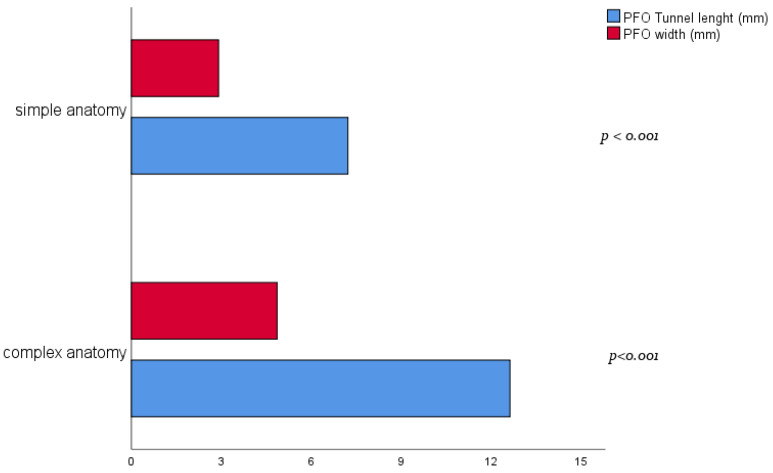
Bar graph with complex and simple anatomy group mean values for PFO width and tunnel length.

The overall population included was found to have a complex anatomy when using conventional parameters and assessing it based on the presence of 1 parameter (a PFO width mean value of 3.8 mm and a tunnel length mean value of 9.8 mm, over 30% with a moderate-to-severe RLS). Therefore, a cluster analysis was performed to differentiate such complexity patterns. Considering both the available echocardiographic evidence and previous literature, the three chosen continuous variables for clustering were the PFO width (mm), tunnel length (mm), and septum secundum thickness (mm). From cluster analysis, 2 clusters were obtained with similar size (54 patients in the first group and 57 in the second one). Comparing the two clusters within the study population, we found no differences in baseline characteristics. However, a significant difference between clusters was found in tunnel length (12.6 ± 3.8 vs. 7.2 ± 2 mm, respectively, *p* < 0.001) and PFO width (4.85 ± 1.8 vs. 2.9 ± 1 mm, *p* < 0.001). At the same time, the septum secundum thickness showed no relevant difference (*p* = 0.99) (Figure 1). Based on the aforementioned features, the first group was identified as “complex anatomy” and the second one as “simple anatomy” (Table 2).

### 3.2. Outcomes and Correlates

**Figure 2:**
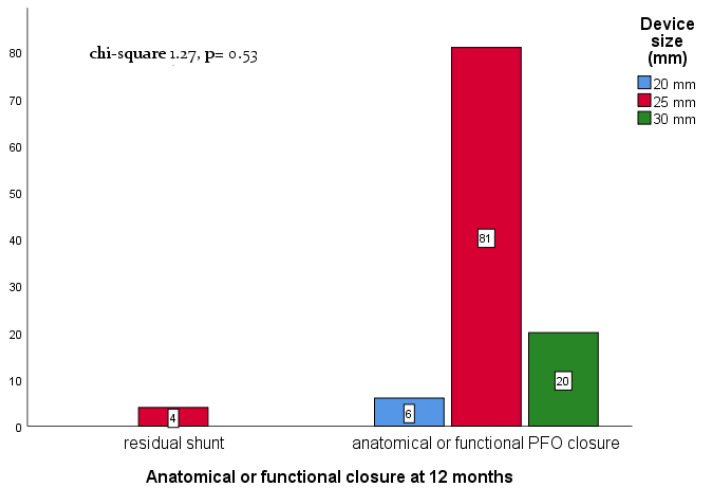
Association of device size and 12-month follow-up transcranial Doppler (TCD) result. The number within the white squares indicates the number of patients for each device subgroup.

At 1-month follow-up, all patients (*n* = 118, 100%) were screened for major events. One death for stroke was reported. Moreover, four paroxysmal atrial fibrillations with spontaneous resolution were reported. The 1-year follow-up was completed in 111 patients (94% of the population); TCD showed an anatomical or functional closure (<10 bubbles residual shunt) in 107 patients (96.4% of patients with complete FU) and residual shunt in 4 (3.6%) (Figure 2). Moreover, 7 patients were lost to follow-up or did not perform a pre-procedural screening for anatomical assessment of the defect. There were no other major adverse events reported at 12 months. At univariable logistic regression analysis, no significant impact of the cluster (complex vs. simple) in the prediction of anatomical or functional closure after 12 months (OR 0.36, CI 95% 0.04–3.6, *p* = 0.39) was found (Table 3). Furthermore, the long-term closure rate is high, regardless of the device size chosen for defect closure (chi-square 1.27, *p* = 0.53) (Figure 2). No association was found between the presence of ASA and rRLS in both clusters (also, one patient with a residual shunt has ASA, and three patients with a residual shunt do not; *p* = 0.67).

## 4. Discussion

The present study reports 1 year of results of a real-world single-center experience of PFO closure performed with a GORE device and the anatomical features of the treated patients in order to understand the correlation between outcomes and anatomical complexity of PFO. The main findings of our study are summarized as follows: (i) PFO closure with a GSO device is feasible and safe in a real-world experience on consecutive patients with a high efficacy (96% of effective closure at 12 months FU) and safety rate (no procedural access-related or device-related complications, no long-term residual damage); (ii) the complexity of the anatomy does not affect the 1-year results.

Percutaneous closure of PFO is a procedure whose main indication is the prevention of cardioembolic stroke recurrence. It is, therefore, a prophylactic intervention and not therapeutic. For these reasons, the maneuver is currently approved and justifiable in cases where it is performed with very high standards of success (efficacy) and minimal incidence of periprocedural complications and “discomfort” for the patient (safety). To date, the PFO procedure is widely available and performed by expert operators only, thus carrying a very low risk of complications (overall rate 2.6% in RCTs) and a high success rate (93–96% of complete closure after 1 year with Amplatzer). A recent position paper has shown an advantage of percutaneous closure over medical treatment [4] and has established clinical indications [14].

Such procedure is considered to be indicated by a mean of multidisciplinary discussion, so the main aim of the accurate TEE evaluation before procedures is to identify the following features: (i) presence of ASA; (ii) thickening of the septum secundum; (iii) morphology and length of the tunnel; and (iv) concomitant presence of interatrial defects or fenestrations of the septum primum, prominent Eustachian valve, Chiari network, and any other finding that might contraindicate the procedure.

One or more of the factors mentioned above could lead to the presence of residual shunt, which has been related to thromboembolic recurrence after PFO closure [15,16]. Therefore, a correction that is as complete as possible is desirable.

Classically, the anatomy of the septum can be distinguished into two categories: simple and complex. The latter is related to a higher rate of the residual shunt [15] and is characterized by the presence of at least one of the complexity factors: the presence of interatrial septal aneurysm (ASA), a long tunnel >8 mm, a wide defects >4 mm, a lipomatous septum secundum with thickness >10 mm and the presence of Chiari’s network or Eustachian valve [10,15]. In the absence of such factors, the septum anatomy is considered “simple”, as in the simple anatomy cluster in our study population. Since only one of the features described above is sufficient to define complex anatomy and considering that no differentiation between the types of complexity has been performed, the results of the published evidence are quite heterogeneous: having a long tunnel is considered as complex as having a thick septum [17]. Several devices are currently available for the treatment of PFO, and they consist of a double disk structure with a larger disk aimed at the right side of the septum and a smaller one on the left side [18,19].

The current devices with the largest experience are the Amplatzer PFO Occluder, STARflex septal closure system, and Gore Septal Occluder. Clinical trials exist for these devices, and few details make each of them more useful in different situations. For instance, APO is an almost rigid device, with a rigid waist not fitting long tunnel morphologies; consequently, in complex anatomies, a high failure incidence was shown [20]. STARflex septal closure system as well as a rigid waist not fitting long-tunnel morphologies; anyway, it has a soft structure limiting erosion. Therefore, complex anatomies are not suitable to be successfully treated with STARflex [6]. The second generation of GSO has been improved; it has a soft waist and structure and has been demonstrated to highly reduce the risk of septal distortion in long-tunnel morphology thanks to its better adaptation to PFO and all the surrounding structures’ anatomy, therefore limiting the risk of erosion as well, as stated elsewhere [21,22]. In this regard, device-induced septal erosion may be linked to higher rRLS incidence, as reported by Cheli et al. in an APO-treated population [13].

As far as the complexity factors are concerned, a huge heterogeneity exists among different studies. Greutmann et al. 2009 [23] found that the presence of ASA in patients undergoing percutaneous PFO closure with an APO significantly increases the rate of residual shunts at 6 months follow-up, even if 35-mm devices are used; no other significant anatomical factors were studied except for right atrium length and Chiari network. Von Bardeleben et al. [6] published the long-term results of a large population of 357 patients treated with three types of devices: Helex, Starflex, and APO. A long follow-up was performed to assess the anatomical and functional closure over time. A significant difference in time of closure was observed in the case of ASA only for Helex and Starflex devices. As for closure time, Lou et al. showed a tight relationship between the effective device’s endothelization and stroke recurrence due to a higher incidence of rRLS in a patients’ group treated by APO with different left/right disc sizes [19].

In addition, the study of Giordano et al. found complex PFO in 25% of the included population; such complexity was defined as PFO width >13 mm, ASA, or multi-fenestrated defect. The study reveals a difference in residual shunt after 12 months in the group treated by a dedicated PFO device [24]. Tunnel length, ASA, and the presence of Eustachian valve were seen as factors defining complex anatomy in the study of Vitarelli et al. [15], where 50% of the included population displayed at least 1 of these criteria and rRLS was found in 12% of the population during FU.

Considering the last generation of GSO devices, Butera et al. published the first early and mid-term multicenter Italian experience; the study did not differentiate the complexity of anatomies of PFO. A very low rate of procedural complication was found, with device malposition in 3% of patients, vascular venous bleeding in 6%, and residual significant shunt in only 3%, of which 1.5% is only more than trivial [8]. Other studies have been published [21], each of them including a small population and not differentiating the anatomical complexity [17,22].

In contrast to previous studies, the overall population of our study presents a complex anatomy with two anatomical factors related to more complex procedures. The anatomy complexity in our population is mainly defined as PFO tunnel length and PFO size, as stated by cluster analysis that identified the first group as more “complex” than the second one. Nonetheless, no difference in outcomes was found between the two groups. In a study by He et al., the presence of a wide PFO (>4 mm) demonstrated an overt impact on RLS severity and rRLS with recurrent stroke [16]. Nevertheless, the mean PFO width of this study population was ≤2.5 mm, different from our population, where a very low incidence of rRLS was reported in both groups with the aforementioned anatomical features (see Table 2).

Therefore, our results are clinically relevant as they confirm a high rate of 12-month anatomical closure at TCD in patients treated with the Gore device, even in complex anatomies. Our study revealed a good device performance as well, in line with what is shown in recent RCT reviews; this was true also regardless of the chosen size [7,8,25]. Our study presents an observational experience, emphasizing the versatility of the device in the high-risk context. Moreover, complex anatomies were not exclusively defined by the presence of ASA or large-shunt but also in terms of several other anatomical characteristics that were not previously taken into account.

In our experience, the presence of ASA was not related to the PFO closure rate at 12 months; this may be due to the low incidence of ASA in the study population (<20%). Moreover, the high stretching capacity of GSO and the related sealing performance ensure a low rate of residual rRLS even in high-risk anatomies (such as those with the association of ASA with PFO). Such stretching capacity may represent the main characteristic that ensures a good performance in the long tunnel defect (>8–10 mm). A retrospective study by Musto et al. shows a head-to-head comparison of GSO and APO in consecutively treated 100 patients with high-risk anatomy. The 1-year closure rate was 96% at transthoracic echocardiography, similar to our study [26]. Nevertheless, in this respect, our paper reports a wider GSO-treated population with the same follow-up period and comparable 1-year closure rate.

### Limitations

This study has some limitations. First, the retrospective nature of the study and the relatively small population size. Although the 1-year FU is among the longest available in the literature, there is a considerable proportion of patients lost in FU, which did not allow us to draw definite conclusions. Furthermore, our patients did not undergo a transesophageal echocardiographic study during follow-up to check for the presence of anatomical closure and/or adverse outcomes (device thrombosis). A dedicated randomized controlled trial for a head-to-head comparison of the GSO with the most used device (APO or StarFlex) is needed to identify the best one in terms of implantation and long-term efficacy and safety. In our study, the absence of a control group for matching could represent a limitation. Our cluster’s elaboration tried to overcome such matters based on anatomical features.

## 5. Conclusions

Our study encourages the GSO as a valid treatment option for high-risk PFO. Unlike the more utilized APO, it has shown a higher versatility that is useful in different anatomical settings. The high closure rate at 1-year follow-up, regardless of the anatomical complexity, underlines the comparable capacity of that device to guarantee long-term outcomes and low complications like the APO. A dedicated post-approval RCT with the GSO is ongoing (Clinical Trial Identifier NCT03821129) with the aim of more validated 12-month results in terms of effective defect closure and stroke recurrences.

## Figures and Tables

**Table 1:** Patient demographic and procedural data.

Patient Population (n)	118
Male gender (n)	60 (50.8%)
Age (years)	55.4 ± 12.7
Current smoker (n, %)	19 (16.1%)
Indication for closure	
Stroke (n, %)	68 (57.6%)
Transient Ischemic Attack (n, %)	50 (42.4%)
Head CT or MRI de-novo lesion (n, %)	101 (85.6%)
TEE Doppler study	
Small shunt (n, %)	15 (12.7%)
Moderate-to-severe shunt (n, %)	37 (31.4%)
Anatomical setting	
PFO only (n, %)	99 (84%)
PFO + ASA (n, %)	19 (16%)
Median procedure time (minutes)	37 (range 33–45)
Median fluoroscopic time (minutes)	7.3 (range 5.5–10.2)
Device used	
20 mm (n, %)	6 (5.0%)
25 mm (n, %)	92 (78%)
30 mm (n, %)	20 (17%)
Median contrast administration (mL)	36 (range 25–54)

Data are presented as mean ± SD, median (IQR), or n (%). PFO: patent foramen ovale; ASA: atrial septum aneurysm.

**Table 2:** Cluster matching for procedural and anatomical characteristics.

	Complex Anatomy (n. 57)	Simple Anatomy (n. 54)	*p*-Value
Age	53.9 ± 12.4	56.6 ± 13.0	0.279
Procedure time (min)	39.6 ± 12.1	41.5 ± 15.2	0.502
Fluoroscopy time (min)	10.1 ± 16.5	9.4 ± 5.8	0.748
Contrast dye (mL)	38.2 ± 26.1	47.1 ± 26.1	0.090
SS thickness (mm)	5.7 ± 2.3	5.7 ± 1.9	0.995
Tunnel length (mm)	12.6 ± 3.8	7.2 ± 2.0	<0.001
PFO width (mm)	4.9 ± 1.8	2.9 ± 1.1	<0.001

Data are presented as mean ± SD.

**Table 3:** Univariable analysis for anatomical or functional PFO closure at 12-month follow-up.

Predictor	Anatomical or Functional Closure at 12-m (%)	OR	C.I. 95%	*p*-Value
Complex vs. simple anatomy *	107/111 (96.4%)	0.36	0.04–3.6	0.39

* For logistic regression, complex anatomy was chosen as reference group. C.I. 95%: confidence interval at 95%, OR: odds ratio.

## Data Availability

The data presented in this study are available on request from the corresponding author. The data are not publicly available due to confidentiality reasons.
